# Napelline in the Treatment of Odontalgia

**Published:** 1886-03

**Authors:** 


### Napelline in the Treatment of Odontalgia.

Grognot (*Bull. gen. de therap*) gives brief notes of a number of cases in which he has used Laborde and Duquesnel's napelline, which, being obtained from *Aconitum napellus*, is to be distinguished from the substance to which Groves gave the same name, but which he extracted from *Aconitum ferox*. The authors' plan was to give a granule of half a centigramme (= about .075 of a grain) by the mouth, every fifteen minutes. Notes are given of six cases, in only one of which did no notable improvement take place. Usually the pain was stopped or greatly mitigated by the time four or five doses had been taken. One of the cases was of two years' standing.—*N. Y. Med. Jour.*

